# Evaluating a novel intervention in undergraduate medicine: an MBBS Curriculum Map

**DOI:** 10.1186/s12909-023-04224-1

**Published:** 2023-04-10

**Authors:** Katie Wardle, Rima Chakrabarti, Tor Wright, Taylor Bennie, Daniel Ntuiabane, Faye Gishen

**Affiliations:** 1 University College London, London, UK; 2 Northern Care Alliance NHS Foundation Trust, Salford, Greater Manchester UK

**Keywords:** Curriculum Development, Curriculum Evaluation, Electronic Curriculum, Digital Learning

## Abstract

**Background:**

Following student feedback, a Curriculum Map (CM) was commissioned in 2018 at UCL Medical School (UCLMS). After exploring key requirements of a CM, the second phase focused on building a prototype before its launch. This study evaluates this novel pedagogical intervention following its implementation, from the perspective of its primary users, UCL medical students.

**Methods:**

This multi-method study was conducted two months after the CM’s launch in 2019. Quantitative and qualitative data was gathered via a survey and focus groups across four domains: usefulness, satisfaction, appearance, and content. Reflective Thematic Analysis was used to analyse the qualitative data to build themes.

**Results:**

One hundred ninety five participants (195/1347, 14%) responded to the survey and two focus groups were held. Higher rates of satisfaction were seen among later years compared to early years students. Five key themes emerged on the CM as a: UCLMS textbook; learning aid for assessments; tool for capturing scientific content; modern learning technology and tool for ‘levelling the playing field’. Key findings suggest that while students welcomed a centralised resource to create transparency, there were clear differences between early and later years students, with the former preferring a more prescriptive approach. Learning was assessment-driven across all years and students highlighted their desire for greater clarity on the importance of curricular content for summative assessments.

**Conclusion:**

A CM provides a benchmark for medical educators on the undergraduate curriculum, which must be balanced with its limitations; a CM cannot provide an exhaustive syllabus and needs to be supplemented with self-directed learning and clinical preparation for practice.

**Supplementary Information:**

The online version contains supplementary material available at 10.1186/s12909-023-04224-1.

## Background

Curriculum Maps (CMs) provide a centralised electronic resource to depict a syllabus and are increasingly being used in undergraduate medical programmes worldwide [1, 2]. CMs promote transparency and have been shown to support fair learning access [3] through outlining and linking Intended Learning Outcomes (ILOs) with assessments and national blueprints, including the General Medical Council’s (GMC’s) *Outcomes for graduates* [4]. CMs also provide opportunities for students to create personalised learning plans and can assist Faculty with timetabling [3].

### UCL Medical School (UCLMS)

**Fig. 1:**
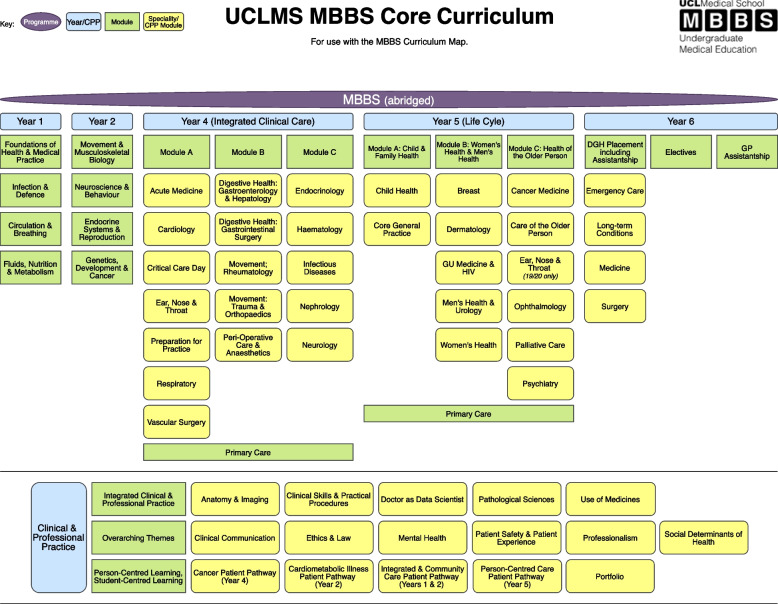
The UCL Medical School MBBS core curriculum during the 2019–20 academic year

Prior to 2019, students on the undergraduate medical (MBBS) programme at UCLMS relied on study guides and Moodle, the virtual learning environment, for accessing the curriculum. Excluding the integrated Bachelor of Science (iBSc) in Year 3; the ‘core’ curriculum consists of seventeen horizontal modules across the six-year programme (see Fig. 1). The first eight modules focus on the fundamentals of scientific practice during Years 1–2 with the remaining modules centred on speciality-based clinical practice (Years 4–6). Sixteen vertical modules feature in Clinical and Professional Practice (CPP) across all years.

**Fig. 2:**
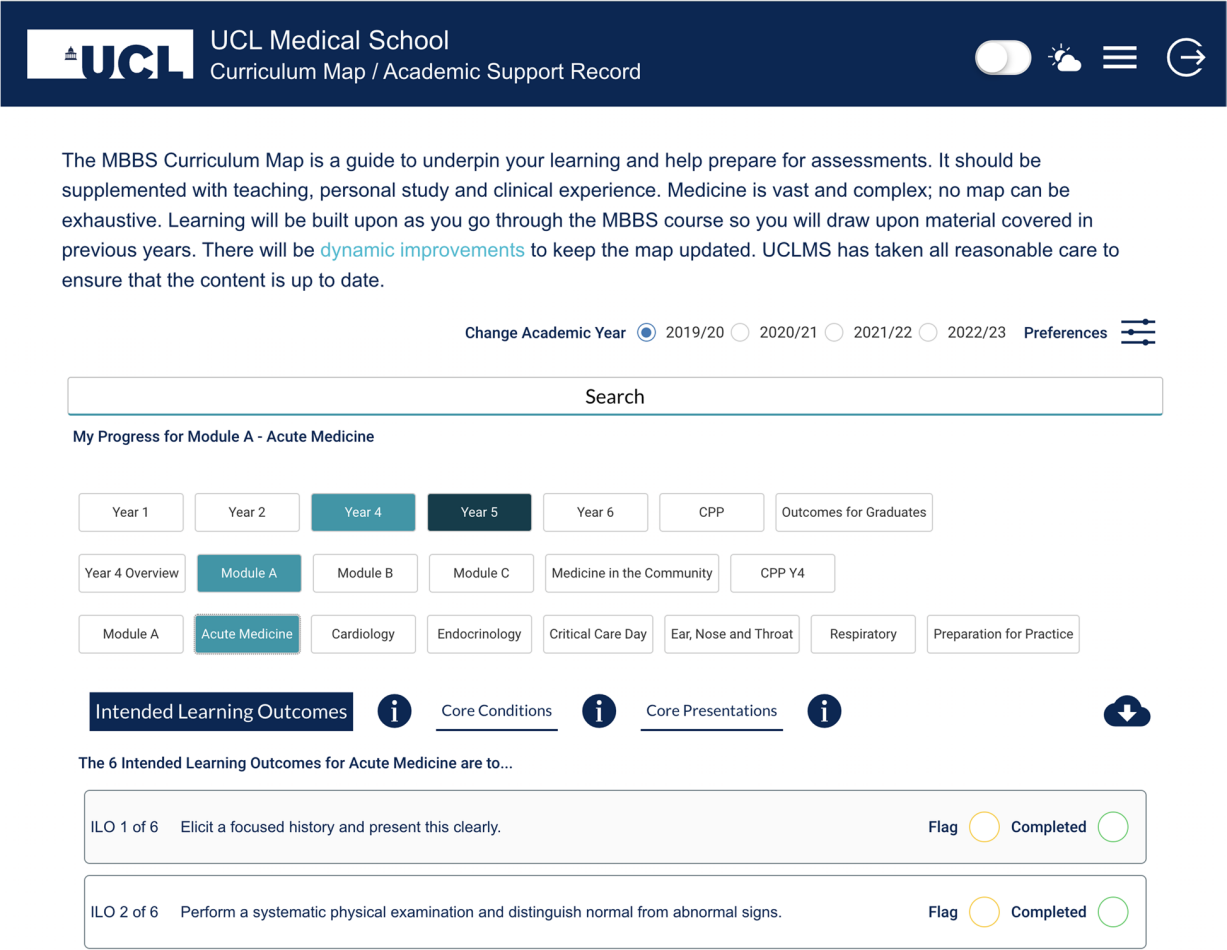
The interface of the 2019-20 MBBS Curriculum Map with the student user selecting Year 4, Module A, and Acute Medicine to show the Intended Learning Outcomes for this specialty

Feedback from the National Student Survey, Staff Student Consultative Committees and Staff Evaluation Questionnaires, revealed that some students felt at a disadvantage due to the lack of clarity around curriculum and assessments [5, 6]. To address this, a Curriculum Mapping Team (CMT) was established in 2018, consisting of an Academic Lead, Clinical Teaching Fellow (CTF), Project Manager and Learning Technologist. The aim was to create and embed a CM at UCLMS in three phases. Phase 1 focused on understanding the requirements of the CM from the perspective of UCL medical students. Five key themes emerged from a survey and focus groups; the need for the CM to be: comprehensive, simple/intuitive, linked throughout the course, aligned to assessments, and enable students to monitor their progress [7, 8]. This initial phase of the study highlighted the importance of setting realistic expectations that the CM would not act as an exhaustive syllabus [8]. Phase 2 of this study focused on building the data structure and design prototype using the software platform bubble.io. To ensure that curricula content was accurately captured, the CMT created customised electronic forms for Faculty to review current data held on ILOs, Core Conditions (CC), Core Presentations (CP) and Sign-off Requirements for each module and specialty. Returned forms were evaluated by the CMT and edited into a house-style to ensure that data had a uniform appearance. Due to a challenging timeframe, Sign-off Requirements could not be finalised for every module and therefore this section was not included in the CM. A final version of each data set was sent to the relevant Faculty members for approval prior to its inclusion within the CM. In conjunction with this, the Learning Technologist collaborated with fourteen students across all years to gather real-time feedback to ensure the design remained student-centred. A final design was created, and the CM was launched in September 2019 for the 2019–20 academic year (see Fig. 2). Year 3 content was excluded from the CM, since the iBSc sits outside of the MBBS programme. Curricular content for Year 6 was also excluded during the pilot year to minimise disruption to students sitting final year summative assessments.

Following on from this work, Phase 3 of the study, and main scope of this paper, was the evaluation of the CM two months after its implementation. Since the CM was theoretically co-designed for a real-world user, the aim of this study was to discover if the CM was ‘fit for purpose’ and identify areas for improvement from the lens of its primary user; UCL medical students.

## Methods

Ethical approval was gained from UCL Ethics Committee. Participation was voluntary and informed; written consent was gained from participants prior to data collection. To explore students’ perceptions of the CM, a multi-method study was undertaken two months after the CM’s launch. During the initial phase, quantitative data was collected through a primary online survey followed by focus group discussions to gain in-depth insight.

### Participants

All Year 1–2 (“early years”) and Years 4–5 (“later years”) UCL medical students were eligible to participate (*n* = 1347). As well as Year 3 iBSc students, Year 6 students were excluded from this study, as in its initial iteration, the CM did not initially include mapping the final year. Information regarding the study was circulated through Moodle where students could register their interest in focus groups and participate in the survey. Students registering their interest were sent a Participant Information Sheet and Consent Form for completion in advance of the focus groups.

### Data collection

Co-designed by the Academic Lead and CTF, the primary survey was created using the software platform Jisc. This consisted of eleven questions covering four domains: utility, appearance, content, and satisfaction of the CM (see Additional file 1). Domains were selected to provide an overall evaluation of the CM following our initial results during Phase 1 of this study [8]. Responses used a four-point Likert scale and an optional free-text response was included to enable participants to add further detail on potential areas of improvement. This free-text response had similarly been used in Phase 1 and had been invaluable in adding insight to the study's findings. All survey responses were anonymous and submitted data was exported to Microsoft Excel for analysis. Free-text responses were grouped into key themes using Reflective Thematic Analysis (RTA) [9].

Two focus groups split between early years (FG 1–2) and later years students (FG 4–5) were conducted (see Table 1). Although focus groups confer the advantage of capturing multiple voices within a shorter timeframe, this must be balanced with group dynamics. It has been recognised that less vociferous participants may encounter difficulties in speaking up [10]. To counteract this, participant numbers were capped between six to ten per group [11]. Each participant was assigned a unique code, identifiable only by gender.

**Table 1:** Focus group participants

Focus group	Total number of participants	Codes
Years 1–2 (early years) group (FG 1–2)	8 (4 male, 4 female)	Male-1; Male-2; Male-3; Male-4; Female-1; Female-2; Female-3; Female-4
Years 4–5 (later years) group (FG 4–5)	10 (5 male, 5 female)	Male-1; Male-2; Male-3; Male-4; Male-5; Female-1; Female-2; Female-3; Female-4; Female-5

To ensure students felt comfortable during discussions, a Year 5 medical student led both focus groups. This student had been trained in focus group facilitation by the Academic Lead with experience leading Phase 1 focus groups. Focus groups followed a pre-determined set of questions covering four domains: functionality, utility, linking to the GMC’s *Outcome for graduates* [4] and future communication (see Additional file 2). Questions were co-designed by the Academic Lead and CTF to explore results from the primary online survey. Focus groups were audio-recorded and transcribed using the professional Rapid Transcriptions service. Each transcript was independently analysed by the Academic Lead and CTF, with the coding framework developed using RTA (see Additional file 3). Data was analysed ‘line by line’ before being grouped into broad themes [9, 12, 13]. To ensure alignment the final framework was agreed between the Academic Lead and CTF.

## Results

One hundred ninety five survey responses were received from 1347 medical students invited to participate (14%). The majority of participants (*n* = 127, 65%) were later years students (see Table 2).

**Table 2:** Survey participants

Group	MBBS Year group	Number of participants	Total
Early years students	1	24	68
	2	44	
Later years students	4	70	127
	5	57	
**All years students**	**Total**	**195**	

**Fig. 3:**
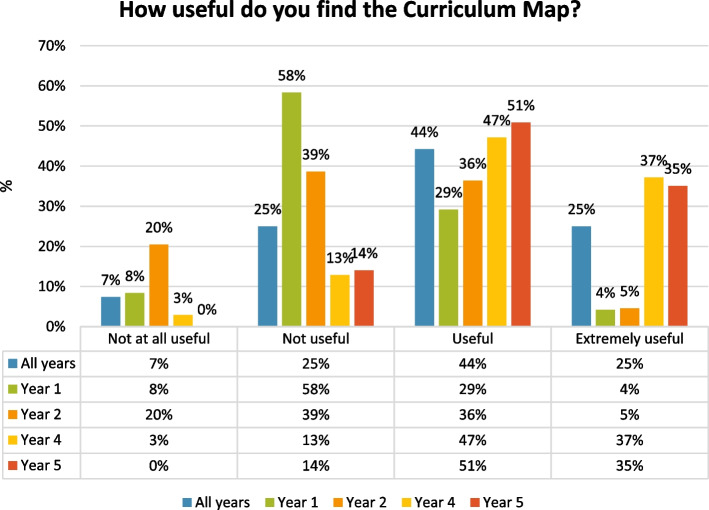
Survey results for: *“How useful do you find the Curriculum Map?”* on a Likert scale from *“not at all useful”* to *“extremely useful”. *Results have been rounded to the nearest whole number

Of the 69% of students that reported that they found the CM either “*useful”* or *“extremely useful”*, the majority were in the later years of the programme (see Fig. 3).

**Fig. 4:**
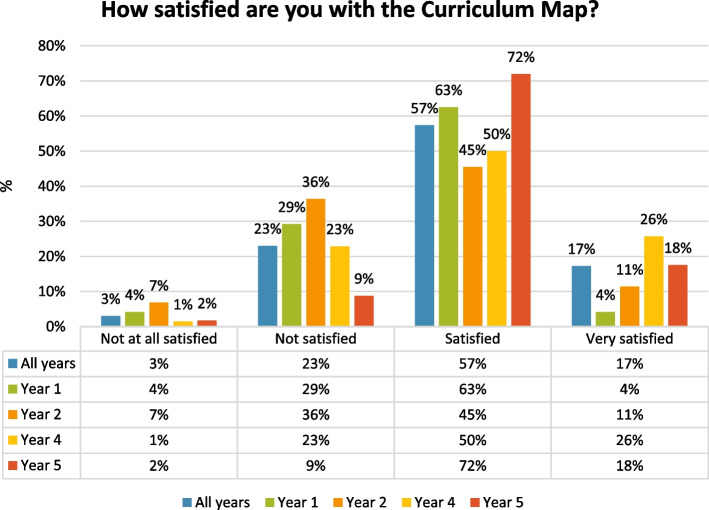
Survey results for: *“How satisfied are you with the Curriculum Map?”* on a Likert scale from *“not at all satisfied”* to *“very satisfied”. *Results have been rounded to the nearest whole number

Similarly, the majority of the 74% of participants who were either *“satisfied”* (57%) or “*very satisfied”* (17%) were later years students (see Fig. 4).

Although 68% of participants felt that curricular content aligned to their teaching, the CM was principally being used for learning related to horizontal modules. Only 6% accessed the CM for CPP-related content. Later years students were more likely to access the CM for CCs (97%) compared to early years students who mostly used it to access ILOs (84%).

86% of participants reported finding the CM “*easy”* or *“very easy”* to navigate and described the appearance of the CM as “*good”* or *“very good”*. 59% identified the ability of *“marking items as complete and seeing the progress bar”* as a useful feature. This was followed by the ability of *“marking items with a flag”* (48%) and *“seeing the links between years/modules”* (40%).

During analysis, there were evident similarities in the themes drawn from both the free-text responses and focus groups. Therefore, five key themes were identified from both data sets with participants regarding the perceived purpose of the CM acting as a:

- UCLMS textbook
- Learning aid for assessments
- Tool for capturing scientific content rather than for soft skills
- Modern learning technology
- Tool for ‘levelling the playing field’ [7]

### UCLMS textbook

There was a clear distinction between expectations of the CM between the two focus groups. While later years students acknowledged that the CM provided a framework on which to base their studies, this was not echoed by early years students:

*“I think this* *[the CM] kind of just gives you the seed and then you can then go and kind of like go off on that what you need to kind of learn from it almost, so it’s quite useful to kind of plant the seed there.” *

*(FG 4-5, Female-5)*

*“We have used the [‘A’ Level] specifications for years now… it’s a very comfortable way of revising…and if [the CM] was going to be something that was a really useful revision resource …it would have to have the format of a specification.”*

                (FG 1-2, Female-6)

While later years students acknowledged the difficulties in having an “*exhaustive list*”, early years students were more in favour of this didactic approach. This may explain why lower rates of usefulness and satisfaction were reported in this group in the survey.

### Learning aid for assessments

There was a sense of anxiety across both groups on the CM’s limitations in relation to assessments. Some participants voiced their scepticism on whether the CM would be used by Faculty when designing assessments:

*“I worry that the curriculum map will not be used by those setting examination questions, therefore information not on the map could come up. The Medical School has not given a guarantee that this won’t happen, therefore I think the map is of limited use.”*

(Free-text response, survey)

Interestingly, participants were in favour of having curricular content stratified by importance to provide more direction in revising for assessments:

*“Maybe having more discriminations between priorities with the objectives to match what is more likely to be examinable (higher ranked priority of the conditions that you think are essential for us to know).”*

(Free-text response, survey)

### Tool for capturing scientific content rather than for soft skills

Across both focus groups, participants highlighted how the CM was principally used for learning *“about the science rather than the soft skills”* (FG 1–2, Male-6). Although participants acknowledged that the CM provided them with context for their portfolio:

*“…the portfolio tab is quite helpful though, because it shows you that there is a reason for what you are doing, because we do get quite a lot of activities and it’s not always clear how they directly relate to like, how we are professionally developing…why what we are doing is important*.”

(FG 1-2, Female-9)

Generally, ILOs related to professional skills were viewed as less amenable to being “ticked off” compared to scientific or clinical content:

*“[Professionalism] is not something that you can measure like that and you just pick it up on the course, and we’re being taught things or like meeting patients or whatever. It’s not really something that’s like a learning outcome as something that you can just go over there and tick off like that.”*

(FG 1-2, Male-1)

Most participants acknowledged that learning soft skills came with clinical exposure and therefore the CM was limited in its use for developing this element of professional practice.

### Modern learning technology

Across both focus groups, the majority of participants reported being satisfied with the CM’s technological features. One advantage described was the ability to access curricular content whenever and wherever. By acting as a centralised resource, the CM enabled participants to track their progress and identify areas for review. Moreover, participants also felt that content was aesthetically pleasing:

“*When you’re looking at the screen, it’s quite nice how clear everything is*.”

(FG 4-5, Female-3)

### Tool for ‘levelling the playing field’

It was highlighted that, given the competitive nature of the MBBS programme, the CM has helped reduce the pressure and anxiety in the lead up to assessments. Participants across both focus groups highlighted how the CM had provided transparency on ILOs. This was recognised as crucial for tackling the perceived inequalities stemming from information-sharing across student societies [5]:

*“Because I feel like…most of us do have friends in the years above and we can ask for advice…but I feel it…just levels the playing field and everyone has got a curriculum that they can refer to and no one feels, like, disadvantaged*.”

(FG 4-5, Female-4)

## Discussion

The CM provides UCL medical students with a framework for the MBBS curriculum, outlining ILOs, CCs and CPs for learning and assessment [3]. The CM was co-developed at UCLMS through a dynamic collaboration with its primary users, and this study provided insight across all years on its purpose and utility within the undergraduate programme. It was clear that medical students felt that the CM had been effective in creating transparency around ILOs, providing all students with a centralised platform to access curricular content. Participants also highly rated additional features, such as tracking their learning progress and writing notes or flagging items for review.

There were some key differences on how useful and satisfied students were with the CM, with higher rates reported among later years students. This had been anticipated, given the findings from the initial stage of the project, where early years students had outlined their preferences for a syllabus similar to their A-Level specifications. In contrast, later years students were more inculcated with self-directed learning and recognised the limitations of a didactic approach. Despite this, most participants were largely in favour of having academic content stratified by importance suggesting that learning remained predominantly assessment-driven across all years. The CMT decided that this call to stratify curricular data by importance would be counterintuitive to the ethos of medical education and the aim of instilling the skills necessary for life-long learning and not just for passing assessments.

While this study provides unique insight into the CM, it is important to acknowledge its limitations. Principally, the data collected is subject to selection bias with only 14% of eligible students participating in the survey and two focus groups conducted. Whilst it is acknowledged that the data collected may not be fully representative of the entire wider student cohort, having two methods of data collection enabled the researchers to gain a more in-depth and richer insight than would have been afforded with just conducting surveys. In addition, the focus groups enabled the key themes identified from the surveys to be explored in further detail.

Whilst the CM depicted the formal curriculum, illustrating the informal or hidden curriculum, which have been recognised for their role in facilitating student learning through observation of key behaviours and practices within the workplace environment, remains a challenge [14, 15].

The findings from our study also highlighted the importance of preparing students for self-directed learning at an early stage of the undergraduate programme and being clear on the purpose of the CM. Essentially, it cannot provide an exhaustive list but rather a framework from which to base their learning from. Interestingly, despite elements of clinical and professional practice being incorporated into the CM, it was recognised that this aspect remained significantly underutilised compared to accessing scientific content among the medical students. Moreover, this study was principally conducted from the perspective of its primary user, medical students, and exploring how other stakeholders, including Faculty members and medical educators, utilise the CM could provide further insight on potential development areas.

This is the first paper, to the authors’ knowledge, that evaluates an undergraduate medicine programme CM following its implementation from the perspective of medical students. Early metrics reinforce students’ views of its value through the 2019 and 2020 National Student Surveys [16, 17] in the domain of Assessment and Feedback, and Student Voice. Additionally, UCL recognised the innovative and technological achievements of the CMT in a Provost Education Award [18].

## Conclusion

This study provided valuable insight on how the CM has helped to create a fairer learning environment within the MBBS programme at UCLMS. Critically it identified that communicating the purpose of the CM and instilling the principles of self-directed learning are crucial to ensure students understand its limitations. This includes how the CM provides a blueprint, rather than an exhaustive list, to aid students’ professional development in becoming a foundation year doctor.

Depicting an entire undergraduate medical programme is no mean feat; Faculty teamwork and a partnership with students is essential to building a student-centred CM. Though the CM is a student-centred resource, this can also be used by educators, to ensure that they are teaching curricular content, and the assessment team, to inform setting assessment questions. The CM promises to be a springboard towards a full MBBS curriculum review at UCLMS. We believe that the CM (subject to revision) offers a viable model that can be used by medical schools to level the playing field for students.

## Supplementary Information

**Additional file 1. **Primary survey questions.

**Additional file 2. **Focus group questions.

**Additional file 3. **Coding framework.

## Data Availability

The data that support the findings of this study are available on request from the corresponding author [KW]. The data are not publicly available due to them containing information that could compromise research participant privacy and consent.

## Abbreviations

CC: Core Conditions
CM: Curriculum Map
CMT: Curriculum Mapping Team
CP: Core Presentations
CPP: Clinical and Professional Practice
CTF: Clinical Teaching Fellow
FG: Focus group
GMC: General Medical Council
ILOs: Intended Learning Outcomes
RTA: Reflective Thematic Analysis
UCLMS: UCL Medical School
