# Supplementary material for: Evaluating a novel intervention in undergraduate medicine: an MBBS Curriculum Map
Source: BMC Med Educ. 2023 Apr 10;23:227. doi: 10.1186/s12909-023-04224-1 (PMC10088241; doi:10.1186/s12909-023-04224-1)
Supplement: Supplementary file 1 — Additional file 1. Primary survey questions. [file 12909_2023_4224_MOESM1_ESM.pdf]

Additional file 1. Primary survey questions

Question	Specific features for inclusion
Which year of the MBBS programme are you in?	• Year 1<br>• Year 2<br>• Year 3<br>• Year 4<br>• Year 5<br>• Year 6
How useful do you find the Curriculum Map?	1 = not at all useful, 4 = extremely useful
How often do you use your Curriculum Map?	• Daily<br>• Weekly<br>• Monthly<br>• Rarely<br>• Never
How do you find the appearance and structure of the Curriculum Map?	1 = very bad, 4 = very good
How do you find navigating around the Curriculum Map?	1 = very difficult, 4 = very easy
Does the content of the Curriculum Map match what you are learning?	1 = never, 4 = always
What do you use the Curriculum Map for? Tick all that apply.	• ILOs<br>• Core conditions<br>• Core presentations (Year 4 and 5 only)
Which of the following features have you found useful? Tick all that apply.	• Marking items with 'flag'<br>• Marking items as 'complete' and seeing the progress bar<br>• Making notes<br>• Creating tags to add to my own notes<br>• Uploading files<br>• Seeing the links between years and modules for core conditions and core presentations<br>• Other (if selected, participants able to specify in a free-text box)
Do you use the map for learning about professional attitudes and behaviours?	• Yes<br>• No<br>• Don’t know
How satisfied are you with the Curriculum Map?	1 = not at all satisfied, 4 = very satisfied
What could we do to improve the Curriculum Map? Please comment below.	
