# Supplementary material for: Evaluating a novel intervention in undergraduate medicine: an MBBS Curriculum Map
Source: BMC Med Educ. 2023 Apr 10;23:227. doi: 10.1186/s12909-023-04224-1 (PMC10088241; doi:10.1186/s12909-023-04224-1)
Supplement: Supplementary file 2 — Additional file 2. Focus group questions. [file 12909_2023_4224_MOESM2_ESM.pdf]

Additional file 2. Focus group questions

Question	Prompts and probes
What do you think about the curriculum map?	What are the advantages of the curriculum map?<br>What are the disadvantages of the curriculum map?<br>How do you find navigating the map?<br>How does the map meet your expectations?<br>Does this match what you're learning?<br>How does it link to other modules/years?
What do you use it for?	What features do you use within the curriculum map?<br>• Monitor progress<br>• Flag/complete<br>• Make notes<br>• Upload files<br>• Creating tags<br>• Searching for items
In the previous survey, the majority of students told us they would like to see links to Outcomes for Graduates. Would you like to see this within the map?	Overview of Outcomes for Graduates (2018) provided by facilitator.<br>Printout shared with participants.
Do you use the map for learning about professional attitudes and behaviours?	
Would you like to receive communications about any changes to the map?	If yes – how? Eg. Moodle page, email, within the map
How would you like to see the Year 6 map? (Years 4-5 focus group only)	Would you like to see a dedicated Year 6 map or be referred to years 4 and 5?
