# Supplementary material for: Evaluating a novel intervention in undergraduate medicine: an MBBS Curriculum Map
Source: BMC Med Educ. 2023 Apr 10;23:227. doi: 10.1186/s12909-023-04224-1 (PMC10088241; doi:10.1186/s12909-023-04224-1)
Supplement: Supplementary file 3 — Additional file 3. Coding framework. [file 12909_2023_4224_MOESM3_ESM.pdf]

Additional File 3. Coding framework

Codes used to analyse data	Themes drawn	Notes
• Local vs national level<br>• Students vs other stakeholders<br>• Aspirations vs practicalities<br>• Learning vs learning for assessment<br>• Hard science vs 'soft' science<br>• Intuition vs metrics<br>• Personalized vs pragmatic<br>• Early years vs later years<br>• Vertical vs horizontal	**Fairness and compromise: *'levelling the playing field'***	• E.g. Outcomes for graduates and the Medical Licensing Assessment vs UCLMS needs<br>• Negotiation compromise<br>• Diplomacy<br>• Reputation<br>• Boundaries<br>• Containment 'I can only do what I can do'<br>• Reminding and constantly refocusing on key questions
• Staying 'true'<br>• Limiting bias, recognising bias (conscious/ unconscious)<br>• Professionalism<br>• Keeping calm!<br>• Loneliness<br>• Bearing disappointment (self, team, colleagues, students)	**The challenges of (leading) change**<br><br>**The burden of conducting insider research**	• 'Us and them'<br>• Taking tough decisions<br>• Retaining 'fresh eyes'<br>• Critical friendship vital (as well as friendship/support)<br>• Accepting 'good enough' perfectionism (see Phases 1&2 of project)

**Codes used to analyse data**	**Themes drawn**	**Notes**
• Control<br>• Leadership<br>• Rigour<br>• Accountability<br>• Good governance		
• Link to Outcomes for graduates (2018)<br>• Curriculum as syllabus (English, Harden, see Theoretical perspectives)	**Negotiating complexity and uncertainty**	• Re-evaluating why I do this job, reflexion, identity, bite-sized chunks e.g. how I break bad news
